# Positive Protective Effects of Sigma-1 Receptor Stimulation with Fluvoxamine after Myocardial Ischemia and Reperfusion in Rats

**DOI:** 10.1007/s11307-025-02030-3

**Published:** 2025-07-03

**Authors:** Xue Zhang, Hiroshi Wakabayashi, Hiroshi Mori, Tomo Hiromasa, Zhuoqing Chen, Takashi Kozaka, Kazuma Ogawa, Seigo Kinuya, Junichi Taki

**Affiliations:** 1 Department of Nuclear Medicine, Kanazawa University Hospital, 13-1 Takara-Machi, Kanazawa, Ishikawa, Japan; 2 Division of Probe Chemistry for Disease Analysis, Research Center for Experimental Modeling of Human Disease, Kanazawa University, 13-1 Takara-Machi, Kanazawa, Ishikawa, Japan; 3 Graduate School of Medical Sciences, Kanazawa University, Kakuma-Machi, Kanazawa, Ishikawa, Japan; 4 Kanazawa Advanced Medical Center, 13-13 Takara-Machi, Kanazawa, Ishikawa, Japan

**Keywords:** Sigma-1 receptor, ^125^I-OI5V, Ischemia, Fluvoxamine

## Abstract

**Background:**

The sigma-1 receptor (Sig-1R) plays diverse roles in regulating Endoplasmic Reticulum (ER) stress, calcium handling, and ion channel activity under pathological conditions, offering cardioprotective effects in pressure overload-induced dysfunction. However, its role in post-myocardial ischemia damage remains unclear. This study evaluated the cardioprotective effects of Sig-1R activation by fluvoxamine following myocardial ischemia in rats.

**Method and Results:**

Wistar rats underwent 20 min of coronary artery occlusion followed by reperfusion. Rats received either saline (control) or fluvoxamine for two weeks. ECG-gated SPECT with ^99m^Tc-MIBI was performed on days 1, 14, and 28 post-reperfusion to measure the end-diastolic volume (EDV), end-systolic volume (ESV), left ventricular ejection fraction (LVEF), and summed rest score (SRS). Autoradiography and histological analyses were performed on day 29. Fluvoxamine significantly improved LVEF after two weeks (D14–D1: 6 ± 7, *p* = 0.03), with the improvement persisting to the 28th day (8 ± 5, *p* < 0.01). Autoradiography revealed a smaller non-salvaged area (0.15 ± 0.19 vs. 0.42 ± 0.32, *p* < 0.05) and more salvaged myocardium (0.33 ± 0.13 vs. 0.14 ± 0.14, *p* < 0.05) in the fluvoxamine group. Histology showed less fibrosis (0.06 ± 0.05 vs. 0.11 ± 0.08, *p* < 0.05) and reduced macrophage infiltration (0.08 ± 0.05 vs. 0.16 ± 0.08, *p* < 0.001) with fluvoxamine.

**Conclusions:**

Sig-1R stimulation by fluvoxamine suppresses LV remodelling and enhances LVEF recovery post-ischemia, suggesting its potential as a novel cardioprotective strategy.

## Introduction

Myocardial infarction (MI), a consequence of an interruption in myocardial perfusion, leads to extensive and often irreversible damage to the cardiac tissue. Timely reperfusion therapy is aimed at limiting acute MI-induced myocardial ischemic injury, thereby improving the patient’s prognosis [1]. However, even after surviving the acute phase, a significant proportion of patients remain predisposed to heart failure, primarily due to adverse geometric, structural, and functional changes in the heart, collectively known as ventricular remodelling [2]. Despite advances in treatment, there is no universally effective drug to prevent myocardial damage that occurs following ischemia–reperfusion.

The Sigma-1 receptor (Sig-1R), an intracellular transmembrane protein primarily located in the endoplasmic reticulum (ER), plays a crucial role in regulating cellular functions at the mitochondria-associated ER membrane (MAM). It functions as a ligand-operated chaperone protein and has an impact on calcium handling, ER stress response, and voltage-gated ion channel modulation [3, 4]. Initially, significant Sig-1R expression was found in the central nervous system, indicating its therapeutic potential for neurological disorders such as Alzheimer’s disease and depression [3, 5].

In recent years, researchers have increasingly focused on the cardioprotective functions of Sig-1R. Studies have indicated that Sig-1R activation reduces cardiac hypertrophy, prevents ventricular remodelling, decreases fibrosis, and improves functional recovery through various pathways that address pressure overload-induced myocardial dysfunction [6–8]. However, to the best of our knowledge, only a few investigations have explored Sig-1R’s role in post-ischemic myocardium.

Our previous studies have evaluated the potential of ^125^I-OI5V as a new tracer to dynamically demonstrate the spatiotemporal expression of Sig-1R. The uptake pattern of ^125^I-OI5V is strongly correlated with the severity of ischemia, peaking three days post-ischemia–reperfusion and progressively declining afterwards [9–11]. In this study, we used ^125^I-OI5V as part of a triple-tracer autoradiographic approach to investigate the protective effects of stimulating the Sig-1R with fluvoxamine, a high-affinity Sig-1R agonist, following myocardial ischemia–reperfusion in rats.

## Methods

### Animal Model of Acute Ischemia–Reperfusion

All experiments adhered to the institutional guidelines set by the Institute for Animal Studies of Kanazawa University. Male Wistar rats (n = 24, 240–260 g, aged 8–9 weeks) underwent left thoracotomy for heart exposure under intraperitoneal anaesthesia (secobarbital sodium, 40 mg/kg) and mechanical ventilation. To occlude the left coronary artery (LCA), a curved needle with a 7–0 polypropylene suture was passed through the myocardium beneath the proximal LCA. The ends of the suture were threaded through a small vinyl tube to form a snare, which was tightened against the tube for 20 min before being released for reperfusion. Myocardial ischemia was confirmed by electrocardiographic ST-segment elevation and localised myocardial cyanosis. The snare was left loosely positioned on the surface of the heart to facilitate LCA re-occlusion just before sacrifice, thereby identifying the area at risk. After the procedure, the rats were randomly divided into two groups for two weeks of intraperitoneal therapy with either saline (the control group) or fluvoxamine at a dose of 10 μg/kg per day (the fluvoxamine-treated group).

### SPECT Imaging for the Assessment of Ventricular Function and Perfusion

Serial ^99m^Tc-MIBI electrocardiogram (ECG)-gated SPECT scans were performed on days 1, 14, and 28 post-ischemia–reperfusion to assess ventricular function and perfusion in the control (n = 13) and fluvoxamine-treated (n = 11) groups. Imaging was performed using a small animal SPECT system (Versatile Emission Computed Tomography, VECTor) equipped with a general-purpose rat/mouse collimator from MI Labs in the Netherlands.

Rats received a tail vein injection of 370 MBq of ^99m^Tc-MIBI 60 min before ECG-gated SPECT imaging to minimise hepatic uptake. Imaging was performed under 2% isoflurane anaesthesia for 15 min. Data were acquired in the list mode, and post-acquisition, photopeak windows (140 keV, 20% width) were applied. Animal studies utilised the triple energy window scatter correction. The cardiac cycles were analysed in 16 frames.

Reconstruction employed pixel-based ordered-subsets expectation maximisation (OSEM) from computed tomography without attenuation correction. PMOD software (PMOD Technologies Ltd., Zürich, Switzerland) was employed for data reconstruction. The voxel size (0.8 × 0.8 × 0.8 mm) was increased ten times to match the human reference heart. The end-diastolic volume (EDV), end-systolic volume (ESV), left ventricular ejection fraction (LVEF), and summed rest score (SRS) were quantitatively assessed using a quantitative gated SPECT software program (QGS, Cedars-Sinai Medical Center, Los Angeles, CA, USA).

### Triple-Tracer Autoradiography

Triple-tracer autoradiography involving ^99m^Tc-MIBI, ^201^Tl, and ^125^I-OI5V was performed 29 days post-reperfusion. Thirty minutes before euthanasia, the Sig-1R imaging tracer ^125^I-OI5V was administered via tail vein injection. Following a 20-min interval, ^201^Tl perfusion tracer (10 MBq) was injected to assess areas of non-viability (non-salvaged area). Ten minutes later, immediately after proximal LCA re-occlusion, 185 MBq of ^99m^Tc-MIBI perfusion tracer was injected to delineate the area at risk (AAR). One minute later, the rat was euthanised, and the heart was harvested for further analysis. The isolated heart was rinsed in saline, embedded in methylcellulose, and sectioned into 20 μm short-axis frozen sections spaced 1 mm apart using a cryostat for autoradiography.

The initial autoradiographic exposure lasted 15–20 min on an imaging plate to visualise ^99m^Tc-MIBI distribution (AAR) within 1–2 h post-sacrifice. Three days later (12 half-lives of ^99m^Tc), a second exposure lasting 5–6 h was conducted to assess myocardial viability indicated by ^201^Tl distribution (salvaged and non-salvaged areas). Finally, a third exposure over seven days imaged Sig-1R expression based on ^125^I-OI5V distribution after one month (10 half-lives of ^201^Tl).

### Autoradiography Data Analysis

Data analysis was performed following the methodology established in a previous study [11]. The digitised autoradiographs were meticulously analysed to map out the distribution of the tracers. Using a sophisticated bioimaging analyser (Typhoon FLA 7000 IP; GE Healthcare, Uppsala, Sweden), the photo-stimulated luminescence intensity from each 50 × 50 μm pixel was precisely quantified. The uptake in each region of interest (ROI) was calculated as the photo-stimulated luminescence per unit area (0.25 mm^2^), adjusted for background levels, to enable rigorous quantitative analyses. The ROI of the background was established adjacent to the left ventricle. The ^99m^Tc-MIBI image was utilised for the manual delineation of the AAR and the normally perfused regions. These delineations were subsequently overlaid onto the ^201^Tl and ^125^I-OI5V images to evaluate the uptake of both tracers. The AAR was divided into salvaged (≥ 60% uptake of normally perfused area) and non-salvaged areas (< 60% uptake of normally perfused area) based on ^201^Tl uptake image. The area ratios of the AAR, non-salvaged area, and salvaged area to the whole LV area, as well as the area ratio of the non-salvaged area to the AAR, and the uptake ratios of ^125^I-OI5V and ^201^Tl in the AAR compared to the normally perfused area were calculated.

### Histological Staining

Short-axis frozen sections of the myocardium (5-μm thick) next to the autoradiography slices were prepared and mounted on slides. These sections were rinsed with phosphate-buffered saline followed by immunostaining with rabbit anti-Cluster of Differentiation 68 (CD68) macrophage antibody (Abcam 125212) to assess macrophage infiltration post-ischemia–reperfusion. Subsequently, secondary immunostaining was conducted using fluorescein isothiocyanate-conjugated goat anti-rabbit IgG H&L (Alexa Fluor® 488) pre-adsorbed (Abcam 150081). Additionally, all nuclei were counterstained with 4′,6-diamidino-2-phenylindole (DAPI; Invitrogen™) for cell density quantification. Hematoxylin–eosin and Masson’s trichrome staining were performed per the standard protocols to assess fibrosis and myocardial structure.

The samples were observed using a fluorescence microscope (BZ-X800, Keyence, Osaka, Japan), and images were recorded using a cooled-charge-coupled device camera. Macrophages in the infarcted area (The infarcted area was determined based on the results of autoradiography) were counted using high-power field (HPF) microscopic images of immunofluorescent labelling. The percentages of CD68-positive and DAPI-positive cells (4 HPF/infarcted area) were computed relative to all DAPI-positive cells within the same HPF in the infarcted region, as well as the area ratio of myocardial fibrosis to the whole LV area, using Image J software (version 1.54d).

### Statistical Analysis

Statistical analyses were conducted using JMP Pro 17. The Shapiro–Wilk normality test was used to assess all quantitative data for normality of distribution. Differences in cardiac function changes between the control and fluvoxamine-treated groups over the observation period were assessed using the paired t-test or the Wilcoxon rank-sum test. Student’s t-test or the Kruskal–Wallis test was employed to compare other parameters between the control and fluvoxamine groups. Linear regression analysis was used to evaluate associations between variables. The threshold for statistical significance was set at *p* < 0.05.

## Results

### Functions of LV by Gated 99mTc-MIBI SPECT/CT

**Fig. 1:**
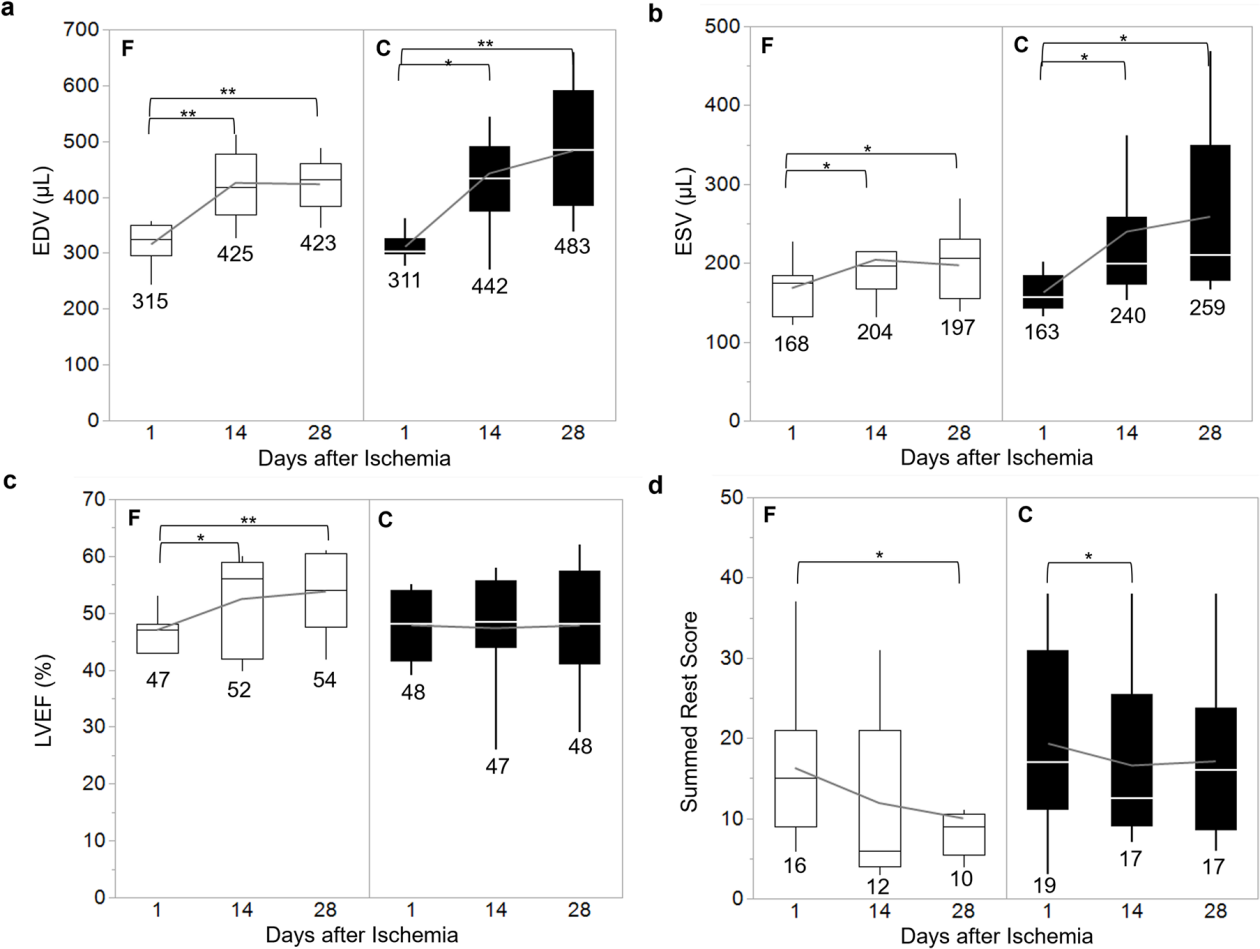
Comparison of EDV (**a**), ESV (**b**), LVEF (**c**), and SRS (**d**) between the Fluvoxamine-treated group (F, white bar) (*n* = 11) and the control group (C, black bar) (*n* = 13) over time (1 day, 14 days, and 28 days post-reperfusion). Significant differences across time points are indicated by *p < 0.05 and **p < 0.01

Fourteen days post-ischemia–reperfusion, a significant increase in EDV was observed in the fluvoxamine-treated (from 315 ± 11.3μL to 425 ± 19.6μL, *p* < 0.01) and control (from 311 ± 6.1μL to 442 ± 34.4μL, *p* < 0.05) groups. A similar trend was noted in ESV, with increments from 168 ± 9.4 μL to 204 ± 17.1 μL in the fluvoxamine-treated group and from 163 ± 6.5 μL to 240 ± 32.6 μL in the control group (*p* < 0.05). Despite these increments, there was no statistically significant difference between the two groups. However, during the subsequent 14 days, even after treatment discontinuation, both EDV (from 425 ± 19.6 μL to 423 ± 15.5 μL, *p* = ns) and ESV (from 204 ± 17.1 μL to 197 ± 15.5 μL, *p* = ns) remained stable in the fluvoxamine group, whereas the control group showed a continuous increase in EDV (from 442 ± 34.4 μL to 483 ± 35.0 μL, *p* = ns) and ESV (from 240 ± 32.6 μL to 259 ± 33.4μL, *p* = ns), although the difference was not statistically significant. (Fig. 1a, b).

As a result, the LVEF in the fluvoxamine-treated group demonstrated continuous improvement after ischemia–reperfusion, particularly within the first 14 days during fluvoxamine administration (from 47 ± 1.9% to 52 ± 2.4%, p < 0.05), while the LVEF in the control group showed almost no change (Figure 1c). SRS decreased progressively in both groups post-ischemia–reperfusion, with a more pronounced reduction observed in the fluvoxamine-treated group (16 ± 2.7, 12 ± 3.3, and 10 ± 2.4 at 1, 14, and 28 days, respectively) compared with the control group (19 ± 3.1, 17 ± 2.9, and 17 ± 3.2 at the same time points; Figure 1d).

### Analysis of the Triple-Tracer Autoradiography

**Fig. 2:**
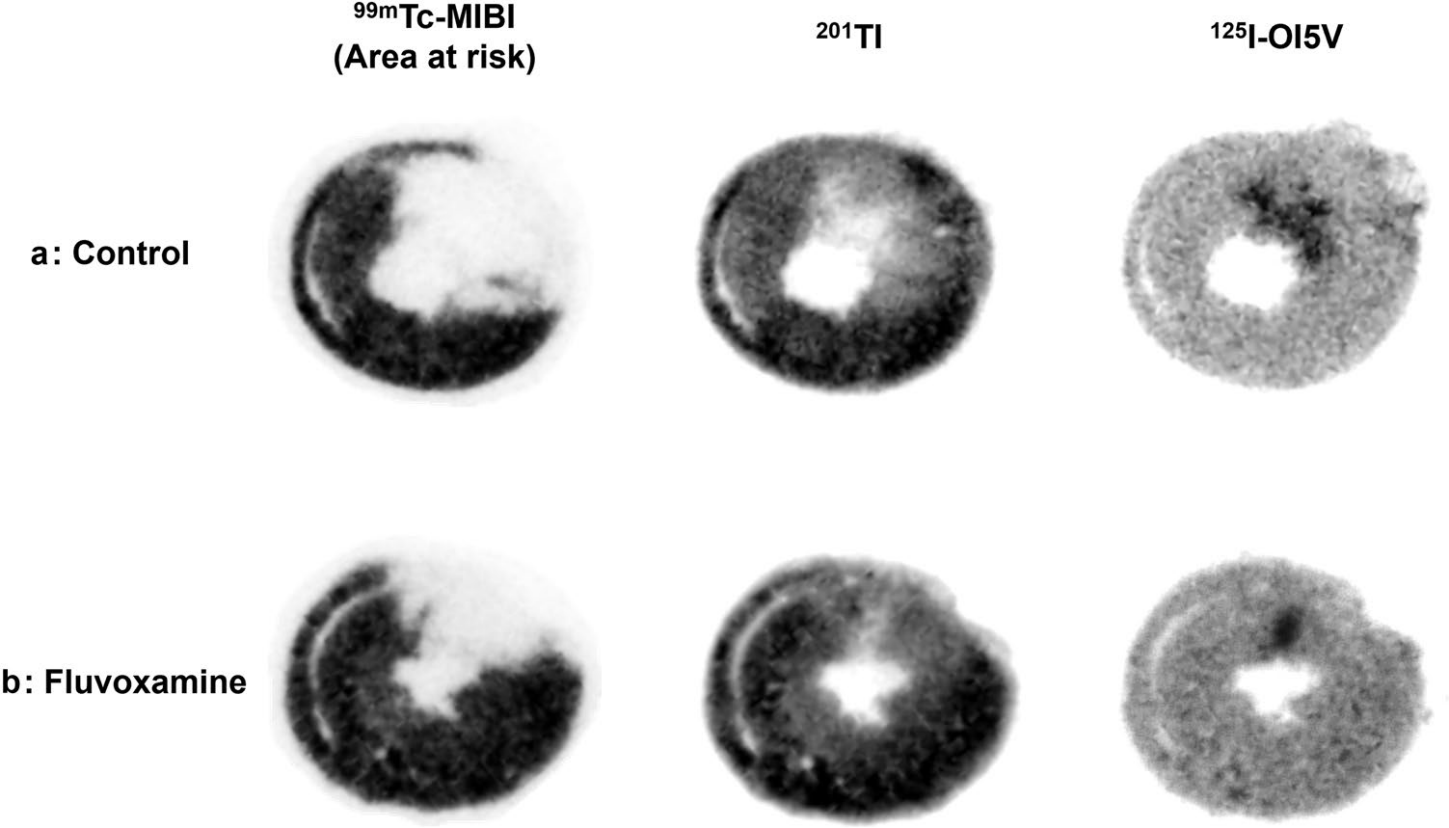
Corresponding triple-tracer autoradiographic (^99m^Tc-MIBI, ^201^Tl, and ^125^I-OI5V) short-axis images in rats 28 days post-ischemia–reperfusion in the control (**a**) and fluvoxamine-treated (**b**) groups. The ^99m^Tc-MIBI and ^201^Tl images demonstrate the area at risk and the non-salvaged area, respectively. ^125^I-OI5V demonstrates the distribution of Sig-1R

**Fig. 3:**
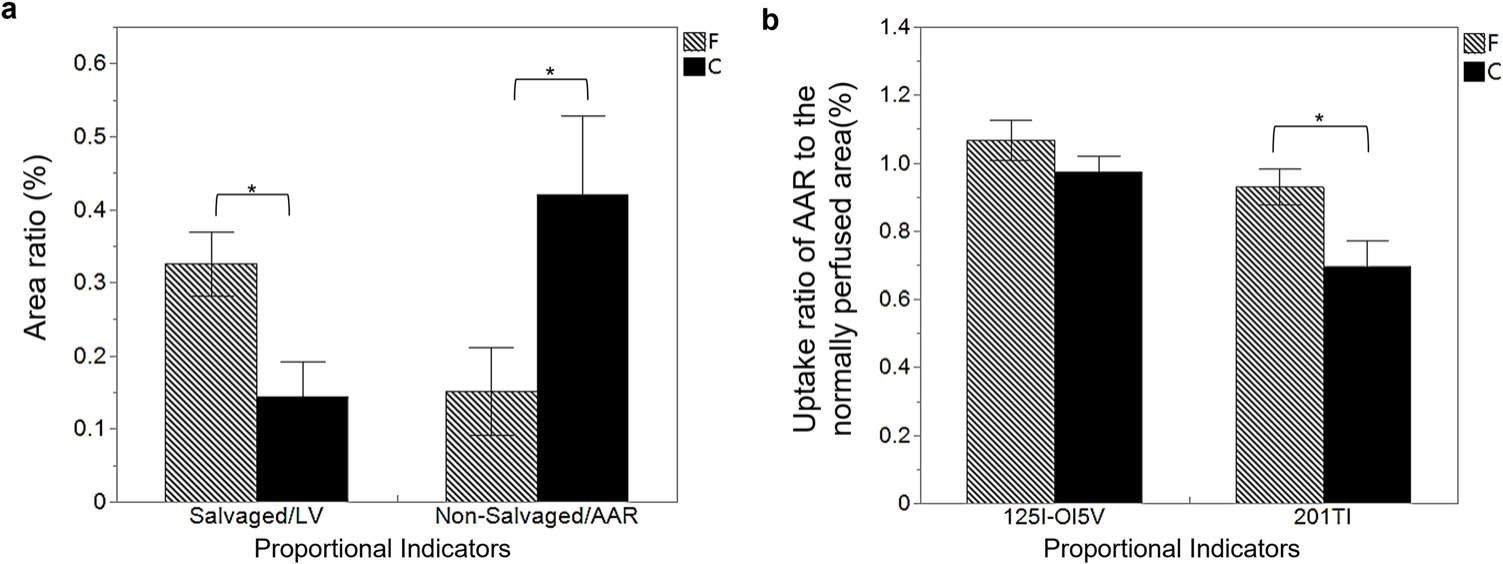
The left panel (**a**) compares the area ratio of AAR/LV, non-salvaged/LV, salvaged/LV, and non-salvaged/AAR between the fluvoxamine-treated (F, hatched bar) (*n* = 9) and control (C, black bar) (*n* = 9) groups. The fluvoxamine-treated group showed a significantly higher ratio of salvaged myocardium (Salvaged/LV, *p < 0.05) and a lower non-salvaged/AAR ratio (*p < 0.05). The right panel (**b**) shows the uptake ratios of ^125^I-OI5V and ^201^Tl, where the fluvoxamine-treated group demonstrates a significantly higher ^201^Tl uptake ratio (*p < 0.05), while ^125^I-OI5V uptake did not differ significantly between the two groups

Figure 2 presents representative triple-tracer autoradiography results for the fluvoxamine-treated and control groups. Autoradiography samples that did not meet the predefined image quality standards were excluded to ensure the reliability of the analysis. A total of n = 9 samples in the fluvoxamine group and n = 9 samples in the control group were included in the final autoradiography analysis. Twenty-eight days after ischemia and reperfusion, both groups exhibited similar AARs; however, the control group had a significantly larger non-salvaged area than the treatment group. Additionally, the ^125^I-OI5V uptake area closely corresponded to the ^201^Tl reduced region. Quantitative analyses indicated that the area ratio of the non-salvaged area to the AAR was significantly lower in the fluvoxamine-treated group (0.15 ± 0.19 vs. 0.42 ± 0.32, *p* < 0.05), while the percentage of the salvaged myocardial area to the whole LV area was significantly higher in the fluvoxamine-treated group than in the control group (0.33 ± 0.13 vs. 0.14 ± 0.14, *p* < 0.05; Fig. 3a).

**Fig. 4:**
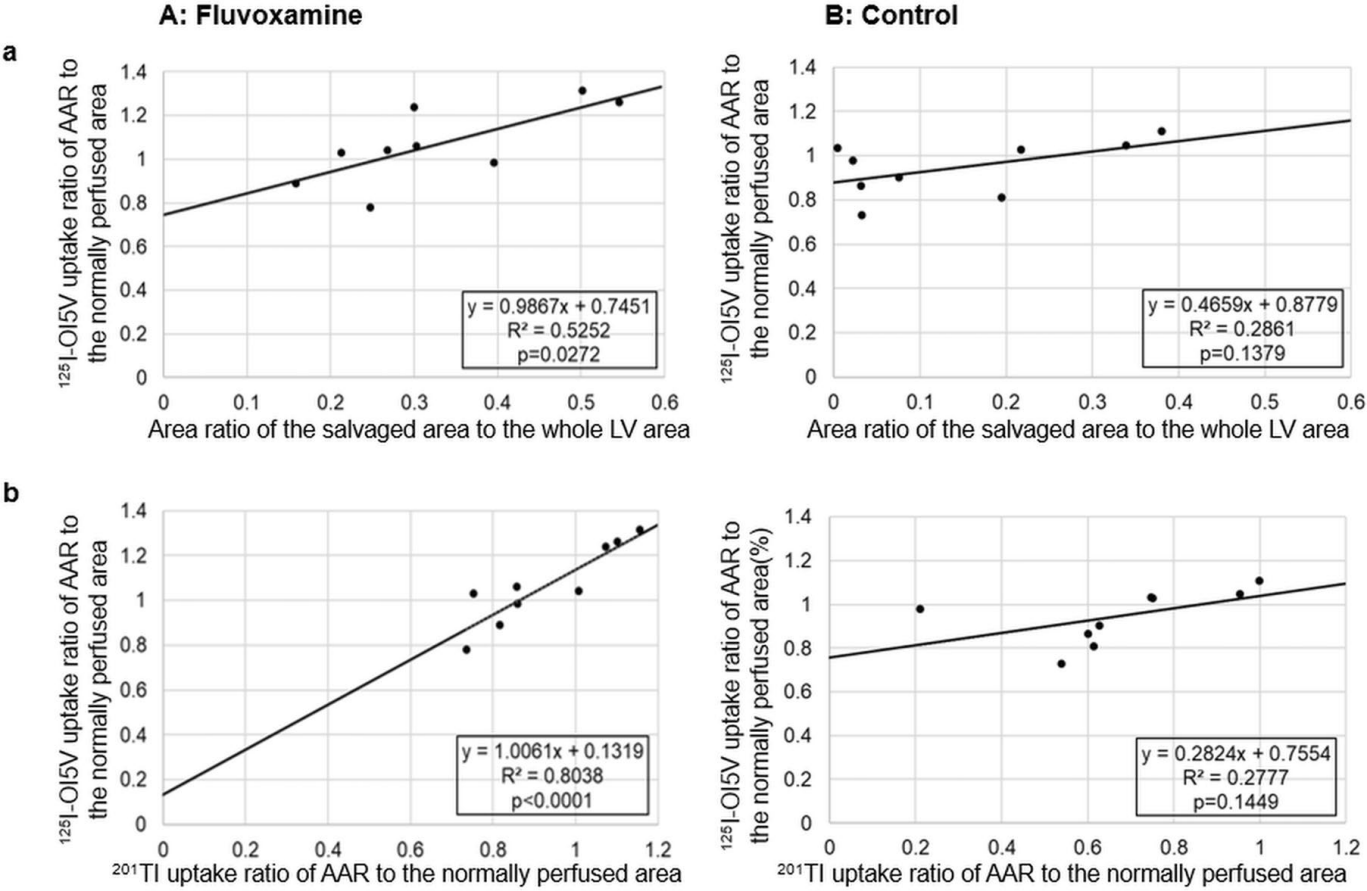
Correlation analysis between ^125^I-OI5V uptake ratio of AAR to the normally perfused area and the area ratio of the salvaged area to the LV area (**a**) and ^201^Tl uptake ratio of AAR to the normally perfused area (**b**) in the fluvoxamine-treated (left) (*n* = 9) and control (right) (*n* = 9) groups. Significant correlations were observed in the fluvoxamine-treated group but not in the control group

No significant difference was observed in the ^125^I-OI5V uptake ratio between the groups (1.07 ± 0.18 vs. 0.95 ± 0.13, *p* = ns). However, fluvoxamine treatment significantly indicated a higher ^201^Tl uptake ratio (0.93 ± 0.16 vs. 0.67 ± 0.23, *p* < 0.05; Fig. 3b). Moreover, in the fluvoxamine group, a positive correlation was found between the ^125^I-OI5V uptake ratio and the area ratio of salvaged area/LV (y = 0.9867x + 0.7451, r = 0.72, *p* < 0.05), and the ^201^Tl uptake ratio (y = 1.0061x + 0.1319, r = 0.89, *p* < 0.01) in the ischemia region. These correlations were not observed in the control group (Fig. 4).

### Histopathological Analysis

**Fig. 5:**
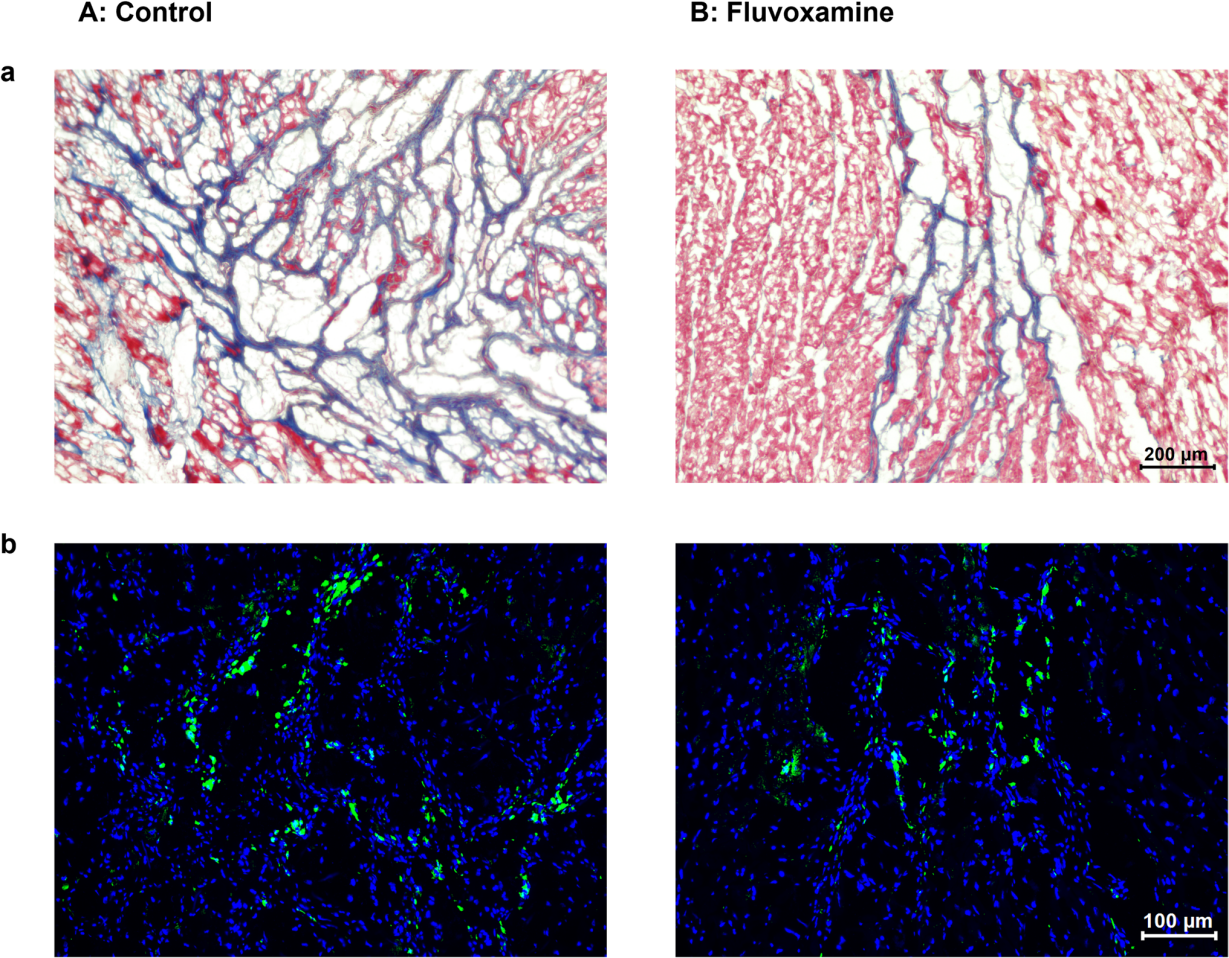
Representative images of histological analyses of myocardial tissue using Masson’s trichrome staining (**a**, Scale bars = 200 μm) and immunofluorescence staining (**b**, Scale bars = 100 μm) in the control (**A**) and Fluvoxamine-treated (**B**) groups 28 days post-ischemia–reperfusion injury. Masson’s trichrome staining revealed greater fibrosis (blue staining) in the control group than in the fluvoxamine-treated group. Immunofluorescence staining revealed a higher infiltration of CD68-positive macrophages (green) with DAPI staining (blue) within the infarcted area in the control group

**Fig. 6:**
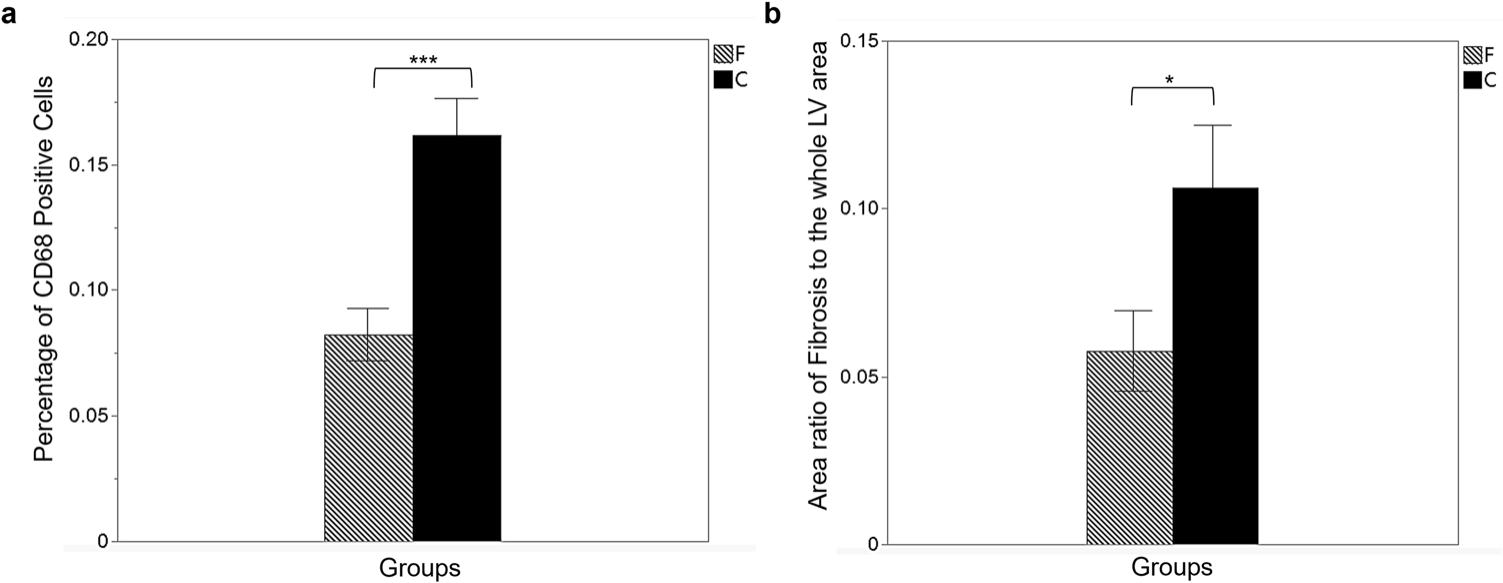
Comparison of the percentage of CD68-positive cells (**a**) and the area ratio of fibrosis to the LV area (**b**) between the Fluvoxamine-treated (F, hatched bar) (*n* = 9) and control (C, black bar) (*n* = 11) groups. The control group exhibited significantly higher fibrosis (***p < 0.001) and macrophage infiltration (*p < 0.05) compared with the fluvoxamine group, indicating the anti-inflammatory and anti-fibrotic effects of Fluvoxamine following ischemia–reperfusion injury

During the sample preparation process, some tissue sections were missing due to incomplete sectioning at the intended cutting level. Consequently, only samples with complete and evaluable sections were included in the final analysis. The final sample size for histopathological evaluation was n = 9 for the fluvoxamine-treated group and n = 11 for the control group. Masson and dual-immunofluorescent staining revealed that a greater portion of the myocardium within the AAR progressed to fibrosis post-ischemia–reperfusion in the absence of fluvoxamine therapy (Fig. 5a), accompanied by significantly increased macrophage infiltration (Fig. 5b). The percentage of CD68-positive cells in the infarcted area was significantly higher in the control group than in the fluvoxamine-treated group (0.16 ± 0.08 vs. 0.08 ± 0.05, *p* < 0.001) (Fig. 6a). Similarly, the area ratio of fibrosis to the whole LV area was significantly greater in the control group than in the fluvoxamine-treated group (0.11 ± 0.08 vs. 0.06 ± 0.05, *p* < 0.05; Fig. 6b).

## Discussion

Recent research has increasingly highlighted the cardioprotective roles of Sig-1R in mitigating hypertrophy, cellular toxicity, apoptosis, and the pressure overload-induced maladaptive endoplasmic reticulum (ER) stress response [12]. Ischemic cardiac injury leads to hypoxia, resulting in a decline in oxidative phosphorylation, reduced cellular adenosine triphosphate (ATP) levels, and the disruption of mitochondrial membrane polarisation. Previous studies have demonstrated that Sig-1R activation mitigated oxidative stress [13, 14], suppressed intracellular Ca^2+^ overload [15], and activated the PI3K/Akt/eNOS pathway [16], potentially protecting against myocardial ischemia-induced injury. Although not many studies have discussed that Sig-1R stimulation can protect the injured myocardium post-ischemia–reperfusion by reducing apoptosis and improving endothelial integrity, Sig-1R agonists were administered before ischemic events in all these studies, potentially confounding the distinction between preventive and therapeutic effects and deviating from clinical reality, thereby reducing the translational relevance of such findings. Moreover, the lack of post-ischemic administration in these studies leaves a significant gap in understanding the true therapeutic potential of Sig-1R activation in a clinical setting, where intervention typically occurs after ischemia has started [17, 18]. To address this limitation and more closely mimic the clinical reality, we started administering fluvoxamine, a well-established Sig-1R agonist, one day after the induction of myocardial ischemia–reperfusion in rats to simulate ischemia and drug administration early after ischemia injury in a realistic context. Our results demonstrated that Sig-1R stimulation by fluvoxamine contributed to LV remodelling suppression and LVEF recovery with myocardium salvage post-ischemia–reperfusion. Furthermore, these cardioprotective effects were sustained even after fluvoxamine was discontinued.

It was observed that in the fluvoxamine-treated group, Sig-1R stimulation resulted in a significantly greater proportion of salvaged myocardium compared with the control group, despite both groups exhibiting similar AAR percentages. The SRS, derived from myocardial perfusion imaging using ^99m^Tc-MIBI ECG-gated SPECT, which quantifies the extent of myocardial perfusion defects at rest, showed improvement in both groups two weeks post-injury, suggesting that the heart’s inherent self-repair mechanisms were at play. However, in the fluvoxamine-treated group, the protective effects persisted for up to two weeks after treatment discontinuation, indicating a sustained therapeutic benefit in mitigating post-ischemia myocardial damage.

Our previous research has established ^125^I-OI5V as a valuable agent for visualising cardiac Sig-1R expression. It has been demonstrated that Sig-1R accumulation occurs particularly in the severely damaged myocardium surrounding non-salvaged areas, peaking around the third day following ischemia–reperfusion and gradually declining by 28 days [9, 11]. Building upon these findings, the current study further highlights the temporal dynamics of Sig-1R activation in mediating cardioprotective effects. Specifically, at 28 days post-ischemia–reperfusion, we observed that the ^125^I-OI5V uptake ratio of the AAR to the normally perfused area correlates positively with the ^201^TI uptake ratio of the AAR to the normally perfused area and the area ratio of the salvaged area to the whole LV in the fluvoxamine group. In contrast, no such correlations were present in the control group. These findings, when considered alongside prior observations of an inverse linear relationship between the ^125^I-OI5V uptake ratio and the ^201^Tl uptake ratio observed three days post-ischemia–reperfusion without intervention [11], suggested that Sig-1R expression initially increased as a compensatory response to ischemia injury, particularly in severely damaged regions. However, without external intervention, this response was insufficient for long-term and sustained cardioprotection, resulting in progressive cell death. By contrast, fluvoxamine administration early after ischemia injury extended the cardioprotective effects of Sig-1R activation, ultimately leading to greater myocardial salvage. Interestingly, many studies that utilised Sig-1R stimulators have reported a significant upregulation of Sigma-1 receptor expression [6–8, 17, 19]. However, in our study, no significant difference in Sig-1R expression was observed. This discrepancy may be attributed to the difference in treatment duration. Unlike studies with continuous drug administration over 28 days, the fluvoxamine treatment in our study was halted 14 days post-ischemia–reperfusion. The natural decline in Sig-1R stimulation post-treatment likely diminished fluvoxamine’s impact, resulting in comparable Sig-1R levels at the 28-day post-ischemia and reperfusion time point.

Initially, M1 proinflammatory macrophages dominate, exacerbating tissue damage through their release of inflammatory cytokines. This phase is followed by the recruitment of M2 anti-inflammatory macrophages, which facilitate tissue repair and contribute to collagen deposition [20, 21]. While the relationship between Sig-1R and macrophage infiltration post-ischemia–reperfusion is not yet fully understood, several studies suggest that Sig-1R plays a key role in neuron-macrophage interactions. For example, the absence of Sig-1R has been shown to reduce macrophage and monocyte infiltration after spared nerve injury, indicating that Sig-1R may influence immune cell recruitment [22, 23]. However, in our study, macrophage infiltration in the myocardium significantly decreased following the administration of fluvoxamine, which contrasts with the findings in the nervous system. These differences could be explained by the tissue-specific role of Sig-1R. In the nervous system, Sig-1R deficiency may impair neuron-macrophage communication by reducing the levels of C–C motif chemokine ligand 2 (CCL2), a key chemokine that mediates macrophage and monocyte recruitment by injured neurons in the dorsal root ganglion (DRG), leading to reduced infiltration of these immune cells [22]. Meanwhile, in the heart, Sig-1R activation likely attenuates inflammation by restoring the activity of protein kinase B (Akt) and endothelial nitric oxide synthase (eNOS) in cardiomyocytes, effectively suppressing macrophage infiltration and polarisation, thereby minimising further tissue damage [24–26]. Our previous study [11] demonstrated that three days post-ischemia, Sig-1R expression closely corresponded to macrophage infiltration. This pattern was corroborated in the present study, where regions with high CD68 expression primarily contained areas of elevated ^125^I-OI5V uptake. This consistent colocalisation across the early and late stages post-ischemia suggests a potential association between Sigma-1 receptor expression and the microenvironment supporting macrophage activation or recruitment. Such distribution may indicate a role for Sig-1R in modulating local inflammation or facilitating tissue repair in the affected areas.

Sig-1Rs are predominantly localized at the MAM, a critical subcellular domain involved in calcium signaling, mitochondrial homeostasis, and redox regulation. This spatial positioning enables Sig-1R activation to mitigate oxidative stress and preserve mitochondrial integrity. In models of pressure overload, Sig-1R activation has been shown to attenuate cardiac hypertrophy and improve contractile function through the Akt-eNOS signaling pathway [7, 27, 28]. Given the overlap in downstream pathological mechanisms, it is conceivable that the cardioprotective effects of fluvoxamine observed in the present study might, at least in part, be mediated through reactivation of this pathway.

Furthermore, evidence from the nervous system indicates that Sig-1R activation enhances Nrf2 signaling and suppresses NF-κB activity [29–31], thereby modulating oxidative and inflammatory responses. Supporting this, independent studies in sepsis models have demonstrated that Sig-1R activation reduces mitochondrial oxidative stress and myocardial injury via the Nrf2/HO-1 signaling axis [32]. Collectively, these findings reinforce the role of Sig-1R as a key regulator of redox homeostasis and cellular protection under pathological stress. Future investigations should aim to elucidate whether these pathways similarly contribute to cardioprotection following ischemia–reperfusion and to explore the therapeutic potential of targeting Sig-1R in ischemic heart disease.

Cardiac fibrosis is a critical pathological repair process observed in various cardiovascular diseases, contributing to heart failure progression, structural deterioration, and, in extreme cases, cardiac rupture [33]. Qu et al. demonstrated the protective efficacy of Sig-1R in cardiac fibrosis using a transverse aortic constriction (TAC) model, showing that Sig-1R offers protection by modulating the activation of cardiac fibroblasts, primarily through IRE1 pathway inhibition and autophagic flux restoration [8]. Consistent with these findings, our study revealed that Sig-1R stimulation significantly reduced the extent of cardiac fibrosis compared with the non-intervention group, highlighting the antifibrotic effects of Sig-1R post-ischemia–reperfusion.

In addition to structural changes, this study demonstrated an improvement in cardiac function. With the administration of fluvoxamine, although the EDV and ESV continued to increase post-ischemia–reperfusion, the rate of this increase was significantly decreased, resulting in a significant improvement in LVEF. In contrast, the control group showed minimal changes in LVEF. Remarkably, this trend persisted for up to two weeks after treatment discontinuation. Similar findings have been reported in other studies conducted using pure MI models with full-course medication, where fluvoxamine administration improved the maximal rate of pressure rise and decline (dP/dtmax and dP/dtmin) and enhanced fractional LV shortening [6, 19].

These findings suggest that fluvoxamine plays a pivotal role in mitigating myocardial dysfunction and preventing adverse remodelling through enhanced myocardial salvage post-ischemia–reperfusion. Furthermore, the prolonged therapeutic effects observed even after treatment cessation highlight its potential for promoting sustained cardiac recovery. These results position fluvoxamine as a promising therapeutic candidate for ischemic heart disease, warranting further investigation to fully elucidate its long-term benefits and underlying mechanisms.

This study has several limitations that are worth mentioning. First, the relatively small sample size may have reduced the statistical power to detect subtle differences between groups; therefore, studies with larger cohorts should be conducted in the future to address this limitation. The reduced sample size was primarily due to mid-experiment mortality and the exclusion of data that showed excessive variability, which could introduce bias. Consequently, more robust experiments with improved consistency will be necessary to investigate the trends observed in this study further. Second, fluvoxamine was administered for only two weeks during the four-week observation period. While this timeframe was chosen based on the hypothesis that short-term interventions could induce sufficient cardioprotective effects, a longer treatment duration might provide additional insights into fluvoxamine’s full therapeutic potential, particularly regarding its long-term effects on cardiac remodelling. The four-week observation period was designed to evaluate infarct healing, typically completed within this period, as well as to capture both the structural and functional changes in the myocardium after treatment cessation. However, a longer observation period could be considered in future studies to better assess the sustained efficacy of fluvoxamine in preventing adverse cardiac remodelling and improving long-term outcomes.

## Conclusions

Fluvoxamine-mediated Sig-1R activation plays a critical role in promoting myocardial salvage and enhancing long-term cardiac function post-ischemia–reperfusion. It also reduced macrophage infiltration and inhibited myocardial fibrosis, which helped to mitigate adverse cardiac remodelling and dysfunction. Furthermore, ^125^I-OI5V imaging served as a valuable tool for evaluating Sig-1R-targeted therapies, enabling the visualisation of receptor accumulation along with its relationship to inflammatory responses and LV remodelling, providing insights into therapeutic efficacy and cardiac injury progression.

## Data Availability

All the data that constitute this study are available from the corresponding author on request.
